# Retraction: Effect of temperature and large guest molecules on the C–H symmetric stretching vibrational frequencies of methane in structure H and I clathrate hydrates

**DOI:** 10.1039/d0ra90048f

**Published:** 2020-05-01

**Authors:** Go Fuseya, Satoshi Takeya, Akihiro Hachikubo

**Affiliations:** Kitami Institute of Technology 165, Koen-cho Kitami 090-8507 Japan hachi@mail.kitami-it.ac.jp; National Institute of Advanced Industrial Science and Technology (AIST) Central 5, 1-1-1, Higashi Tsukuba 305-8565 Japan

## Abstract

Retraction of ‘Effect of temperature and large guest molecules on the C–H symmetric stretching vibrational frequencies of methane in structure H and I clathrate hydrates’ by Akihiro Hachikubo *et al.*, *RSC Adv.*, 2018, **8**, 3237–3242, DOI: 10.1039/c7ra12334e.

We, the authors, hereby wholly retract this article due to the incorrect calibration of temperature in the Raman measurements. This incorrect calibration means that the temperature values for Raman spectroscopy shown in the article were inaccurate, and the actual temperature range was higher than the range reported in the original article.

Having consulted with an independent expert, the Royal Society of Chemistry has determined that any changes made to the paper to correct this would be major, and therefore that the best course of action is retraction and republication of the article with the correct data. The Royal Society of Chemistry is happy that the overall conclusions of the paper are not affected by this error, and therefore that republication of the work with the correct data is appropriate. The republished article can be found at https://doi.org/10.1039/D0RA02748K.

We, the authors, brought this matter to the attention of the Royal Society of Chemistry ourselves, and are happy with the decision to retract and republish this article.

Signed: Akihiro Hachikubo (on behalf of the authors)

Date: 15^th^ April 2020

Retraction endorsed by Laura Fisher, Executive Editor, *RSC Advances*

## Supplementary Material

